# Improving clinical management of macular neovascularisation secondary to angioid streaks

**DOI:** 10.1038/s41433-023-02797-1

**Published:** 2023-11-28

**Authors:** Godhuli Patri, Ibtesam Elaroud, Nicholas Beare, Savita Madhusudhan

**Affiliations:** 1 St. Paul’s Eye Unit, Royal Liverpool University Hospital, Prescot Street, Liverpool, L7 8XP UK; 2 Department of Eye and Vision Sciences, University of Liverpool, William Henry Duncan Building, 6 West Derby Street, Liverpool, L7 8TX UK

**Keywords:** Retinal diseases, Vision disorders

Angioid streaks (AS) occur due to breaks in a degenerated and weakened Bruch’s membrane and typically radiate outwards from the optic disc. They could be idiopathic, or associated with systemic diseases such as pseudoxanthoma elasticum (PXE), Ehler–Danlos syndrome, Paget’s disease, Sickle cell disease, and other hemoglobinopathies [1]. Macular neovascularisation (MNV) is a common cause of central visual loss and occurs in 42–86% of cases, with bilateral involvement in up to 71% [2].

We would like to share with the readers a protocol defining management of AS-related MNV following a review of our clinical practice for patients referred between 2014 and 2022 to St. Paul’s Eye Department, Royal Liverpool University Hospital, for either active management or second opinion. We identified patients with secondary MNV for analysis. Data pertaining to patient demographics, management of MNV, and visual outcomes was retrospectively collected from electronic patient records.

Of the 24 patients with AS, 18 (10 males; 31 eyes) had MNV. The average age of these patients was 57.9 years, 55.6% had a diagnosis of PXE; 72.2% had bilateral MNV. Mean follow-up was 48 months (4 months–13 years). The location of MNV was subfoveal, juxtafoveal, and extrafoveal in 32%, 32%, and 36% of eyes, respectively. Details of anti-VEGF intravitreal therapy were available in 21 out of 31 (67.7%) eyes. Twelve eyes had aflibercept, 9 received ranibizumab initially followed by aflibercept. The mean number of injections per eye was 20.6, reducing to 16.7 (range 2–45) when excluding one patient who had 89 injections. The mean best-recorded logMAR visual acuity at baseline and final follow-up was 0.57 and 0.50, respectively. Overall, 52% of eyes gained, 29% lost and 19% maintained baseline visual acuity at the final follow-up. One incidence of endophthalmitis with recovery to baseline visual acuity was noted. At final follow-up, central retinal thickness on macular OCT was lower in 12 eyes (57.2%), remained stable in 8 eyes (38%), and was worse in 1 eye (4.8%) compared to baseline.

**Fig. 1:**
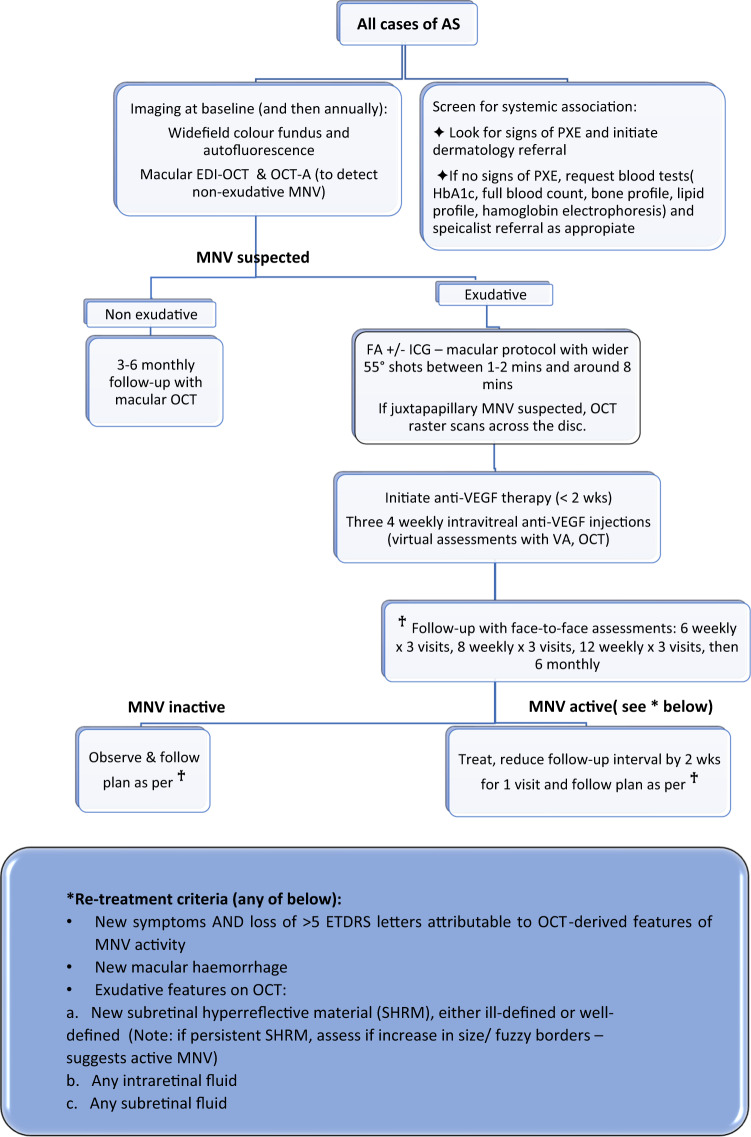
St. Paul’s Eye Department AS-related MNV management protocol. EDI enhanced depth imaging, OCT-A ocular coherence tomography angiography, SHRM subretinal hyperreflective material, VEGF vascular endothelial growth factor, HbA1C haemoglobin A1c.

Although our visual outcomes are comparable to published studies [3] with different anti-VEGF agents using *p.r.n*. regimes, our patients had received a higher average number of injections per eye (Table 1). Given the lack of established guidelines to inform treatment decisions for AS-related MNV, we devised a departmental protocol to aid clinical decision-making that may be more widely applicable (Fig. 1).

**Table 1:** Comparison of different studies on management of MNV in AS.

Study	Anti-VEGF	Regime	No. of eyes	Age (years), mean	Follow-up (months), mean	No. of injections, mean	Visual acuity			Assessment	Outcome measures	CRT reduction, mean
							Improved	Stable	Reduced			
Sawa et al. [4]	Bevacizumab	PRN	15	59	19	4.5	5 (33%)	8 (54%)	2 (13%)	BCVA, OCT, FA	BCVA, FA	NA
Elmatri et al. [5]	Bevacizumab	PRN	18	NA	12	4.8	11 (61.1%)	7 (38.8%)	0 (0%)	BCVA, OCT, FA	BCVA, CRT	103 u
Teixeira et al. [6]	Bevacizumab	PRN	5	36	25	5.8	5 (100%)	0 (0%)	0 (0%)	BCVA, OCT, FA, ICG	BCVA, CRT, FA	69 u
Finger et al. [7]	Bevacizumab	PRN	16	55	28	6.5	13 (81%)	0 (0%)	3 (19%)	BCVA, OCT, FA, ICG	BCVA, CRT, FA	39.8 u
Wiegand et al. [8]	Bevacizumab	PRN	9	70.8	19	4.4	4 (44.4%)	4 (44.4%)	1 (11.1%)	BCVA, OCT, FA,	BCVA, CRT	67.7 u
Bhatnagar et al. [9]	Bevacizumab	PRN	9	53.5	6	1.8	2 (22%)	7 (78%)	0 (0%)	BCVA, OCT, FA	BCVA, CRT	161 u
Ladas et al. [10]	Ranibizumab	PRN	15	58.9	12	7.1	8 (53.3%)	6 (40%)	1 (6.7%)	BCVA, OCT, FA	BCVA, CRT, FA	107.1 u
Mimoun et al. [2]	Ranibizumab	PRN	35	63.7	24.1	5.7	4 (11.4%)	26 (74.3%)	5 (14.3%)	BCVA, OCT, FA	BCVA, CRT	NA
Vadala et al. [11]	Ranibizumab	PRN	9	58	14	5	7 (78%)	1 (11%)	1 (11%)	BCVA, OCT	BCVA, CRT	46 u
Shah and Amaoku [12]	Ranibizumab	PRN	12	NA	21.75	5.75	3 (25%)	8 (66.67%)	1 (8.33%)	BCVA, OCT	BCVA, CRT	38.8 u
Tilleul et al. [13]	Ranibizumab	PRN	35	63.2	48.6	9.9	5 (14.3%)	17 (48.6%)	13 (37.1%)	BCVA, OCT, FA	BCVA, CRT, FA	NA
Grenet et al. [14]	Ranibizumab	PRN	98	55.5	36.5	4.1	3 (15.8%)	10 (52.6%)	6 (31.6%)	BCVA, OCT or FA	BCVA, CRT, FA	46 u
Gliem et al. [15]	Aflibercept	PRN	15	53	12	6.7	4 (26.7%)	10 (66.7%)	1 (6.6%)	BCVA, OCT, FA, retinal sensitivity	BCVA, CRT, FA, retinal sensitivity	38 u
Our study	Ranibizumab + Aflibercept	PRN	21	57.9	48	16.7	11 (52.3%)	4 (19%)	6 (28.5%)	BCVA, OCT, FA	BCVA, CRT	67.9 u

*BCVA* best-corrected visual acuity, *CRT* central retinal thickness, *OCT* ocular coherence tomography, *FA* fluorescein angiography, *ICG* indocyanine green.

Although anti-VEGF therapy can stabilise or even improve visual outcomes in AS-related MNV, MNV can be tenacious with a high recurrence rate, often requiring long-term anti-VEGF therapy, as seen in our case series. Our results are affected by the retrospective nature of the review and a high degree of subjectivity in re-treatment decisions by clinicians. We have defined precise re-treatment criteria as part of the management, which we believe will prevent under or over-treatment and aim to re-audit our results in future. Given the limitations of our small sample size, further prospective studies with larger sample sizes and longer follow-up duration are required to support our conclusions.
